# *Tropheryma whipplei* Endocarditis Diagnosed by Tissue 16S rRNA Gene Sequencing: A Case Report

**DOI:** 10.1016/j.cjco.2025.10.008

**Published:** 2025-10-25

**Authors:** Takashi Maeda, Shun Kohsaka, Nozomi Niimi, Shunsuke Uno, Yoshifumi Uwamino, Mika Nagata, Yasuyuki Shiraishi, Yoshikazu Kishino, Masaki Ieda

**Affiliations:** a Department of Cardiology, Keio University School of Medicine, Tokyo, Japan; b Department of General Internal Medicine, National Hospital Organization Tokyo Medical Center, Tokyo, Japan; c Department of Infectious Diseases, Keio University School of Medicine, Tokyo, Japan; d Department of Laboratory Medicine, Keio University School of Medicine, Tokyo, Japan; e Clinical Laboratory, Keio University Hospital, Tokyo, Japan

**Keywords:** blood culture-negative endocarditis, *Tropheryma whipplei*, 16S rRNA gene sequencing

**A 66-year-old man with arthralgia and fever of unknown origin developed progressive aortic and mitral regurgitation with vegetations. Despite a staged and extensive diagnostic work-up over 6 months, no pathogen was identified. Ultimately, valve replacement surgery was performed, and 16S rRNA gene sequencing of the excised valve tissue confirmed the presence of *Tropheryma whipplei*. The patient was successfully treated with ceftriaxone and long-term trimethoprim-sulfamethoxazole. *T*. *whipplei* is a rare cause of infective endocarditis that poses a diagnostic challenge. This case highlights the critical role of molecular diagnostics in identifying fastidious organisms in patients with culture-negative endocarditis.**

## Case Presentation

A 66-year-old man presented with intermittent fevers and weight loss of approximately 8 kg over 10 months. Three years before this presentation, he had been evaluated by a physician for arthralgia in multiple joints, including the ankles, knees, wrists, and elbows. The cause of the polyarthralgia was presumed to be either gout or fever-related pain, and he was treated conservatively with nonsteroidal anti-inflammatory drugs (NSAIDs). At that time, frequent premature ventricular contractions were observed, and transthoracic echocardiography (TTE) demonstrated mild aortic and mitral regurgitation. He had no other symptoms and was followed up conservatively.

Laboratory tests showed no neutrophilia or leukocytosis and only a mild elevation in inflammatory markers, with C-reactive protein levels ranging from 11.6 to 72.6 mg/L. ^18^F-fluorodeoxyglucose positron emission tomography and computed tomography (FDG PET-CT) revealed no signs of an infectious source, malignancy, or other causes of fever. Prior to 6 months, subsequent TTE demonstrated progression of valvular disease with moderate-to-severe mitral regurgitation and multiple vegetations on the mitral valve. Although 14 sets of blood cultures were performed, no causative pathogens were identified. The patient presented with no identifiable epidemiologic risk factors typically associated with blood culture-negative endocarditis (BCNE), such as recent antibiotic exposure prior to blood culture, injection drug use, contact with infected livestock, or exposure to soil, animals, body lice, or cats. As the patient remained stable, empirical antibiotics were discontinued. Subsequently, the patient was referred to our hospital for diagnostic examination and surgical valve replacement.

**Figure 1:**
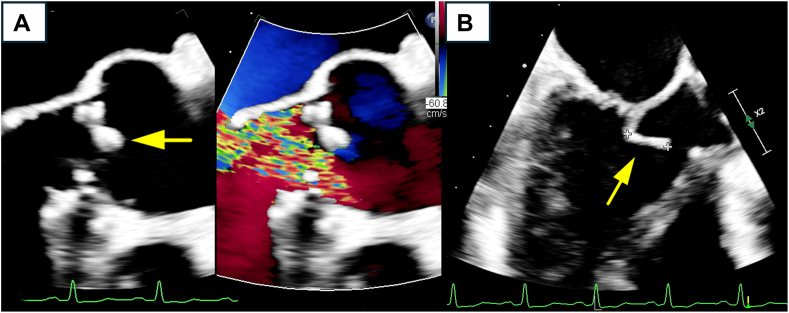
(**A**) Transesophageal electrocardiography long-axis 2-dimensional view of the aortic valve. The **yellow arrow** indicates a thickened, highly echogenic area on the noncoronary cusp. The color Doppler demonstrates severe eccentric regurgitation. (**B**) Transesophageal electrocardiography long-axis 2-dimensional view of the mitral valve. The anterior leaflet of the mitral valve is globally thickened. The **yellow arrow** indicates a mass measuring approximately 12.2 mm at its tip. The mass appears to protrude into the left ventricular outflow tract during systole.

At admission, the patient appeared comfortable, without respiratory distress. His vital signs were as follows: blood pressure, 136/52 mm Hg; pulse, 95 beats per minute; body temperature, 36.3 °C; and respiratory rate, 16 breaths per minute. Physical examination revealed a diastolic murmur at the third left sternal border (grade III/VI). The remainder of the examination findings were unremarkable, with no evidence of Osler nodes, Janeway lesions, Roth spots, or other peripheral signs of endocarditis. Laboratory tests demonstrated an essentially normal complete blood count, metabolic panel, and liver function, with a mildly elevated C-reactive protein level (15.1 mg/L). TTE and transesophageal echocardiography revealed severe aortic regurgitation and moderate mitral regurgitation, along with multiple vegetations on the mitral valve, the largest of which measured 18 × 9 mm (Figure 1, Video 1, and Video 2).

**Figure 2:**
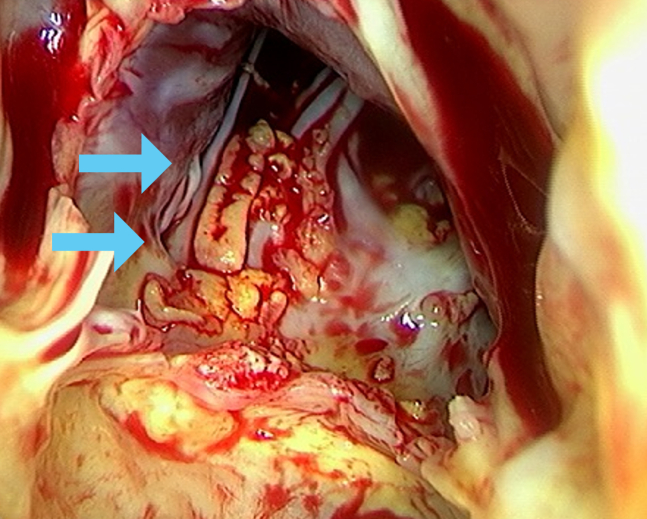
Intraoperative findings of the mitral valve from the left ventricular side. Multiple vegetations are observed on the anterior leaflet of the mitral valve (**light blue arrows**).

Despite a thorough and continued workup for BCNE, including prolonged culture incubation and antigen tests for *Coxiella* spp., no causative organisms were identified. Due to the gradual progression of valve regurgitation and an increase in the size of the vegetation, a decision was made to proceed with double valve replacement. After multidisciplinary discussions with cardiac surgeons and infectious disease specialists, we decided to withhold preoperative empirical antibiotic therapy to prioritize the identification of the causative pathogen from the valvular tissue. This decision was based on the patient’s stable vital signs and imaging findings, which demonstrated no evidence of an embolism on systemic computed tomography or brain magnetic resonance imaging. A single dose of prophylactic cefazolin was administered immediately before surgical incision. Surgical pathology revealed that the aortic valve was free of vegetation, whereas the anterior leaflet of the mitral valve showed abundant vegetations on the left ventricular side (Figure 2). Histopathologic examination of these specimens demonstrated fibrosis, myxoid degeneration, and mild inflammation, with no definitive evidence of infection.

Postoperatively, ceftriaxone and doxycycline were administered to provide empirical coverage for native valve endocarditis, including the potential *Haemophilus, Aggregatibacter, Cardiobacterium, Eikenella,* and *Kingella* (HACEK) organisms, as well as *Bartonella* and *Coxiella*. However, cultures from the surgical specimens remained negative after 10 days. In consultation with the microbiology laboratory, the specimens were sent for 16S rRNA gene sequencing to identify fastidious, difficult-to-culture pathogens. On postoperative day 15, sequencing revealed a *Tropheryma whipplei*-specific rRNA gene. The antibiotic regimen was adjusted to 4 weeks of ceftriaxone followed by 1 year of oral trimethoprim-sulfamethoxazole (TMP-SMX). The patient’s fever and arthralgia resolved. The patient was discharged on postoperative day 40. Follow-up echocardiography confirmed well-functioning prosthetic valves, and the patient remains relapse-free for 3 years postoperatively.

## Discussion

Here, we report a rare case of culture-negative endocarditis caused by *T. whipplei*. *T. whipplei* is found in the environment, particularly in human-associated settings, such as sewage and wastewater, with most carriers remaining asymptomatic.^1^ Only a subset develops significant clinical syndromes such as Whipple’s disease, which primarily affects the joints and gastrointestinal system, causing weight loss, diarrhea, and malabsorption.^2^ Although *T. whipplei* classically has been considered a rare cause of cardiovascular disease, it is now increasingly recognized as an essential etiology of infective endocarditis, owing to increased recognition and advances in diagnostic molecular techniques, such as 16S rRNA gene sequencing on blood or vegetation.^3^ The appropriate duration of treatment for infectious endocarditis caused by *T. whipplei* is more than 1 year, highlighting the importance of identifying the pathogen using modern techniques.

Current clinical practice guidelines suggest using molecular biology techniques, such as 16S rRNA gene sequencing of blood or excised valve tissue, to identify causative pathogens in patients with BCNE.^4^ Indeed, rapid advances in sequencing-based diagnostics have improved the ability to identify pathogens in infective endocarditis. Although polymerase chain reaction (PCR) analysis of duodenal biopsy specimens is considered a useful extracardiac diagnostic tool when Whipple’s disease is suspected, this case highlights the particular value of using molecular techniques on cardiac valve tissue itself.^1^ Specifically, 16S rRNA gene sequencing of valvular biopsies has demonstrated greater sensitivity and specificity than conventional blood cultures.^5^^,^^6^ In Japan, 16S rRNA gene sequencing is not widely available due to a lack of public health insurance coverage. Such coverage and the widespread adoption of modern diagnostic techniques are crucial for enhancing the quality of care for infectious diseases caused by rare and fastidious pathogens. This case highlights the importance of modern molecular approaches for the diagnosis of BCNE.

Administering empirical antibiotics before pathogen identification poses a clinical dilemma: it can hinder definitive microbiologic diagnosis, yet it is often necessary to stabilize critically ill patients.^4^ Prior antibiotic administration is a key contributor to BCNE.^3^ Further, long-term preoperative antibiotic therapy reduces the sensitivity of molecular biological tests on excised valve tissue.^7^ Therefore, prioritization of pathogen diagnosis may still be necessary, taking into account the patient's overall condition and symptoms of embolism. In the modern era of molecular diagnostic testing, research on the appropriate timing of empirical antibiotic therapy for BCNE is required.

## Conclusion

In the modern era, molecular biology techniques are crucial for pathogen identification, enabling the development of effective treatments. The clinical course of our case, spanning over 2 years, underscores the importance of recognizing early manifestations (eg, arthralgia and gastrointestinal symptoms), which can point to rare infectious etiologies, such as *T. whipplei*. Finally, advances in sequencing techniques, including 16S rRNA gene sequencing, have significantly improved the detection of fastidious pathogens, emphasizing their value in clinical practice.

**Novel Teaching Points**

• *T. whipplei* can cause fever of unknown origin and BCNE.
• Molecular analyses, such as 16S rRNA gene sequencing, are critical for identifying fastidious organisms, such as *T. whipplei.*
• Prioritizing pathogen diagnosis may be necessary, considering the patient’s general condition, symptoms of embolism, and the possibility of long-term antibiotic administration.

## Supplementary Material

Video 1 TEE revealing severe aortic regurgitation before surgery.

Video 2 TEE revealing severe mitral regurgitation with vegetations on the mitral valve before surgery.
